# Obstetric and newborn outcomes of mothers with and without HIV infection in Anaka general hospital in Northern Uganda

**DOI:** 10.1371/journal.pone.0326322

**Published:** 2025-06-17

**Authors:** Jolly Joe Lapat, Jimmyy Opee, James Okello, Nixson Oyoo, Christopher Wanican, Gloria Becky Labong, Daniel S. Ebbs, Felix Bongomin

**Affiliations:** 1 Department of Public Health, Faculty of Medicine, Gulu, Uganda; 2 Anaka General Hospital, Nwoya District Local Government, Gulu, Uganda; 3 Department of Reproductive Health, Faculty of Medicine, Gulu University, Gulu, Uganda; 4 Kitgum General Hospital, Kitgum District Local Government, Kitgum, Ugnada; 5 Section of Critical Care Medicine, Department of Paediatrics, Yale University, New Haven, Connecticut, United States of America; 6 Department of Medical Microbiology and Immunology, Faculty of Medicine, Gulu University, Gulu, Uganda; 7 Department of Internal Medicine, Gulu Regional Referral Hospital, Gulu, Uganda; Debre Markos University, ETHIOPIA

## Abstract

**Background:**

Women of reproductive age constitute a significant proportion of the global HIV burden, with millions becoming pregnant while on lifelong antiretroviral therapy (ART). Although ART has dramatically improved maternal and child health outcomes, concerns persist regarding its safety in pregnancy. This study compares obstetric and neonatal outcomes between women with and without HIV infection at Anaka General Hospital, a rural hospital in northern Uganda.

**Methods:**

A hospital-based retrospective cross-sectional study at Anaka General Hospital in northern Uganda was conducted from July 2020 to June 2023. A total of 914 delivery records were included, sampled using systematic random sampling from the hospital maternity register. Data were extracted from maternity and neonatal records using a structured tool. Associations between HIV status and obstetric or neonatal outcomes were assessed using Chi-square or Fisher’s exact tests for categorical variables and independent sample t-tests for continuous variables. Multivariable logistic regression was used to adjust for potential confounders and a p < 0.05 was considered statistically significant.

**Results:**

Of the 914 participants included in the study 38 (4.2%) were HIV-positive. The odds of congenital anomalies were significantly higher among infants born to HIV-positive women compared with HIV-negative women (adjusted odds ratio = 9.76, 95% confidence interval: 1.72–55.48, p < 0.01). No significant differences were observed in maternal or neonatal outcomes between the two groups.

**Conclusion:**

HIV infection was significantly associated with an increased risk of congenital anomalies, while other obstetric and neonatal outcomes were similar between HIV-positive and HIV-negative women. We recommend enhanced prenatal monitoring and early fetal screening among HIV-positive pregnant women on dolutegravir-based ART. Additionally, prospective studies are needed to better understand the contribution of dolutegravir and maternal factors to congenital anomalies in HIV-exposed pregnancies.

## Introduction

Women of reproductive age account for more than half of the 39 million people living with human immunodeficiency virus (HIV), globally [1]. The majority of these women are of childbearing age and are capable of transmitting the infection to their babies. Maternal transmission accounts for over 90% of infections in children under 15 years of age. For more than two decades, the World Health Organization (WHO) and UNICEF have recommended lifelong anti-retroviral therapy (ART) for all pregnant women living with HIV, regardless of CD4 + cell count [2]. This policy has enabled many women with HIV to live longer, healthier lives and to conceive while on ART. Each year, an estimated 1.5 million women living with HIV give birth [3].

According to the Joint United Nations Programme on HIV/AIDS (UNAIDS) 2023 global report, between 64 and 98% of pregnant women with HIV globally have access to ART [1]. ART has significantly reduced maternal morbidity and mortality [1,4,5]. However, concerns remain regarding pregnancy outcomes in HIV-positive women compared to their HIV-negative counterparts. Research has shown that women living with HIV are 2–3 times more likely to experience adverse pregnancy outcomes due to direct effects of HIV, complications of ART, and associated socioeconomic vulnerabilities [6–8]. Specific complications include preterm labour [7,9], maternal anemia [10], puerperal sepsis [11,12], post-partum hemorrhage [11–13], intrauterine growth restriction (IUGR) [14], and a higher likelihood of cesarean delivery [14,15]

Neonates born to HIV-positive mothers are also at increased risk of adverse outcomes including congenital anomalies [16–18], stillbirths [17,19], low Apgar scores [7,9] low birthweight [20], Neonatal Intensive Care Unit (NICU) admission [21], and higher neonatal mortality [7,19]. Although ART has proven effective in preventing mother-to-child transmission (MTCT) and improving maternal health [1,12], certain ART regimens particularly those including zidovudine (AZT) and dolutegravir (DTG) have been linked to an increased risk of congenital anomalies [22,23].

While the benefits of ART in reducing MTCT and improving maternal health are well-established, potential associations with adverse pregnancy outcomes possibly influenced by socioeconomic status and rural residence highlight the need for further investigation. There is a lack of data on maternal and neonatal outcomes among women with HIV in Uganda.

This study aimed to address this gap by comparing maternal and neonatal outcomes among women with and without HIV infection who gave birth at Anaka General Hospital in Northern Uganda. Specifically, the study conducted in 2024 assessed whether HIV infection was associated with an increased risk of adverse obstetric and neonatal outcomes in this rural setting.

## Materials and methods

### Study design and setting

This study was a hospital-based retrospective records analysis of deliveries conducted at the maternity ward of Anaka General Hospital, Nwoya District, in Northern Uganda from July 2020 to June 2023. Anaka General Hospital is the only government-owned hospital under governance of Nwoya District Local Government And serves as the major referral facility for the district. The clientele includes patients from Nwoya as well as neighboring districts with an average of 2000 deliveries conducted annually. All pregnant mothers in labour and those with pregnancy complications are admitted to the maternity ward, which also houses a Neonatal Intensive Care Unit for managing sick newborns (NICU). All HIV positive women are enrolled in care at the Mother Baby care clinic at first ANC contact, and viral load counts are evaluated every three months. CD4 + tests are not routinely collected and clients are initiated on a DTG containing regimen according to policy [24].

### Study population

The study population consisted of all deliveries from Anaka General Hospital between July 2020 and June 2023. We excluded charts of women who delivered before arrival and twin pregnancies due to their higher risk of adverse outcomes.

### Sample size estimation

The sample size was calculated using the Kish Leslie formula, incorporating the doubling proportion formula [25]. By utilizing a critical value of 1.96 at a significance level of 5%, and considering a margin of error of 0.05 along with a statistical power of 80%, a sample size of 914 was deemed adequate for detecting a minimum odds difference of 2.0 in low birth weight compared to a previous study [8].

### Sampling strategy

During the study period, there were a total of 6,662 deliveries. We excluded 167 charts due to twin deliveries or births before arrival. The final sampling frame included 6,495 eligible deliveries. Systematic random sampling was used to select records from the integrated maternity register. The sampling interval (K) was calculated as 6,495 divided by the required sample of 914, yielding a K value of approximately 7. The first chart was selected using simple random sampling, after which every 7^th^ record was included. The sample was proportionately allocated between HIV-positive and HIV-negative women according to their distribution in the delivery population.

### Data collection

The collection process, encompassing both records retrieval and data extraction, was conducted from January 3 to February 29, 2024. Data were collected from maternal records using a structured data extraction tool developed by the research team. The tool was pretested and structured to capture both maternal and neonatal outcomes. Two midwives, trained by the principal investigator, served as research assistants. They were briefed on the study objectives, data collection procedures, and proper use of the tool. Sample charts identified from the integrated maternity register were listed with patient names and in-patient numbers. Charts of sampled mothers and babies admitted to the NICU were retrieved from the records section. Data were then extracted from these files. The authors had no access to identifiable personal information during or after data collection.

### Study variables

Our outcome variables were grouped as obstetric or newborn. The obstetric outcome variables included mode of delivery (categorized as vaginal delivery or caesarean section), preeclampsia, defined as hypertension and proteinuria (++) using a urine dipstick, ante partum haemorrhage, episiotomy performed, perineal tear, defined as any tear that required repair, post-partum haemorrhage and maternal death. The newborn outcome variables included delivery status (alive or dead), birth weight, Apgar score at 1 and 5 minutes, presence of congenital anomalies, admission to NICU and neonatal death.

The data extraction tool captured data on independent variables including socio-demographic characteristics and HIV status. The main socio-demographic factors included age, area of residence and referral status. Medical factors captured focused on HIV status. The obstetric factors included gravidity, parity, gestational age at delivery, stage of labour at admission and onset of labor.

### Data management and analysis

The raw data was thoroughly checked for accuracy and completeness before being coded and entered into an Excel spreadsheet. Analysis was conducted using STATA software version 17 SE, with all tests being two-tailed at a 95% confidence interval and significance level set at p < 0.05. Baseline characteristics of participants were summarized, with the corresponding p-values indicating distribution disparities between cohorts. The association between categorical variables and HIV status was analyzed using Chi-square test or Fisher’s exact test where appropriate. Association between numerical variables and HIV status was assessed using the independent student’s t-test. Level of statistical significance was set at p < 0.05. Outcome variables that had significant association with HIV status were taken to multivariate logistic regression to adjust for other independent variables. Results are presented as adjusted odds ratio (aOR) with the corresponding 95% confidence interval and *p –* values (Table 1–3 - 3).

**Table 1:** Comparison of socio-demographics characteristics.

Characteristics	Total N (%)	With HIV n (%)	Without HIV n (%)	P value
**Age** (mean, sd)	22.2	26.0	22.0	**< 0.001**
**Address**				
Urban	421 (46.2)	15 (39.5)	406 (46.5)	0.395
Rural	490 (53.8)	23 (60.5)	467 (53.5)	
**Parity**				**0.001**
Multiparous	441(48.3)	28 (73.7)	413 (47.2)	
Nulliparous	473 (51.7)	10 (26.3)	463 (52.8)	
**Referral in**				0.786
Yes	109 (11.9)	4 (10.5)	105 (12.0)	
No	805 (88.1)	34 (89.5)	771 (88.0)	
**Labor onset**				0.919
Induced	26 (2.9)	1 (2.6)	25 (2.9)	
Spontaneous	370 (97.1)	37 (97.4)	833 (97.1)	
**Stage of labor**				0.743
Not in labor	111 (12.1)	6 (15.8)	105 (12.0)	
Latent phase	250 (27.4)	8 (21.1)	242 (27.6)	
Active phase	412 (45.1)	17 (44.7)	359 (45.1)	
Second stage	141 (15.4)	7 (18.4)	134 (15.3)	

**Table 2:** Comparison of obstetric outcomes.

Variable	Total N (%)	HIV Positive n (%)	HIV Negative n (%)	P value
**Mode of delivery**				0.245
Caesarean	153 (16.7)	9 (23.7)	144 (16.4)	
Vaginal	761 (83.3)	29 (76.3)	732 (83.6)	
**Indication of C/S**				0.234
Prolong/obstructed	38 (25.2)	1 (11.1)	37 (26.0)	
Fetal distress	19 (12.6)	1 (11.1)	18 (12.7)	
Previous C/S	45 (29.8)	6 (66.7)	39 (27.5)	
Contracted pelvis	25 (16.5)	1 (11.1)	24 (16.9)	
Pre-eclampsia	9 (6.0)	0 (0.0)	9 (6.3)	
Others	15 (9.9)	0 (0.0)	15 (10.6)	
**Type of caesarean**				0.412
Elective	10 (6.6)	0 (0.0)	10 (7.0)	
Emergent	142 (93.4)	9 (100.0)	133 (93.0)	
**Pre-eclampsia**				0.619
Yes	24 (2.6)	0 (0.0)	24 (2.7)	
No	890 (97.4)	38 (100.0)	852 (97.3)	
**Ante-partum hemorrhage**				0.410
Yes	11 (1.2)	1 (2.6)	10 (1.1)	
No	903 (98.8)	37 (97.4)	866 (98.9)	
**Obstructed/prolonged**				0.516
Yes	31 (3.4)	2 (5.3)	29 (3.3)	
No	882 (96.6)	36 (94.7)	846 (96.7)	
**Perineal tear**				0.979
Tear	119 (13.0)	5 (13.2)	114 (13.0)	
No tear	795 (87.0)	33 (86.8)	762 (87.0)	
**Episiotomy**				0.521
Performed	44 (4.8)	1 (2.6)	43 (4.9)	
Not performed	870 (95.2)	38 (97.4)	833 (95.1)	
**Post-partum hemorrhage**				1.00
Yes	12 (1.3)	0 (0.0)	12 (1.4)	
No	902 (98.7)	38 (100.0)	864 (98.6)	
**Puerperal sepsis**				1.00
Yes	1 (0.1)	0 (0.0)	1 (0.1)	
No	456 (99.9)	38 (100.0)	875 (99.9)	
**Hospitalization days** (median, range)	2 (1, 18)	2 (1, 8)	1 (1, 18 )	0.433

**Table 3:** Comparison of neonatal outcome.

Variable	Total N (%)	HIV positive n (%)	HIV negative n (%)	P value
**Status**				0.719
Stillbirth	17 (1.9)	1(2.6)	16 (1.8)	
Live birth	897 (98.1)	37 (97.4)	860 (98.2)	
**Apgar 1 minute**				0.924
< 7	77 (8.4)	3 (7.9)	74 (8.5)	
7–10	836 (91.6)	35 (92.1)	801 (91.5)	
**Apgar 5 minute**				0.967
< 7	25 (2.6)	1 (2.6)	24 (2.7)	
7–10	889 (97.4)	37 (97.4)	851 (97.3)	
**Birth asphyxia**				0.660
Yes	92 (10.3)	3 (8.11)	89 (10.4)	
No	805 (89.7)	34 (91.9)	771 (89.6)	
**Birth weight**	2.9	2.76	2.93	0.032
**Anomaly**				**0.003**
Yes	8 (0.9)	2 (5.3)	6 (0.7)	
No	905 (99.1)	36 (94.7)	869 (99.3)	
**NICU admission**				0.370
Yes	104 (11.59)	6 (16.2)	98 (11.4)	
No	793 (88.41)	31 (83.8)	762 (88.6)	
**Discharged alive**				0.895
No	44 (4.81)	2 (5.3)	42 (4.8)	
Yes	870 (95.84)	36 (94.7)	834 (95.2)	

### Ethical statement

Ethics approval was obtained prior to the study’s initiation. The Gulu Research Ethics Committee granted approval for the study (Ref GUREC-2023–586), including a waiver of consent. Additionally, administrative clearance was obtained from the Chief Administrative Officer of Nwoya District Local Government to proceed with the research.

## Results

### Study enrollment

**Fig 1:**
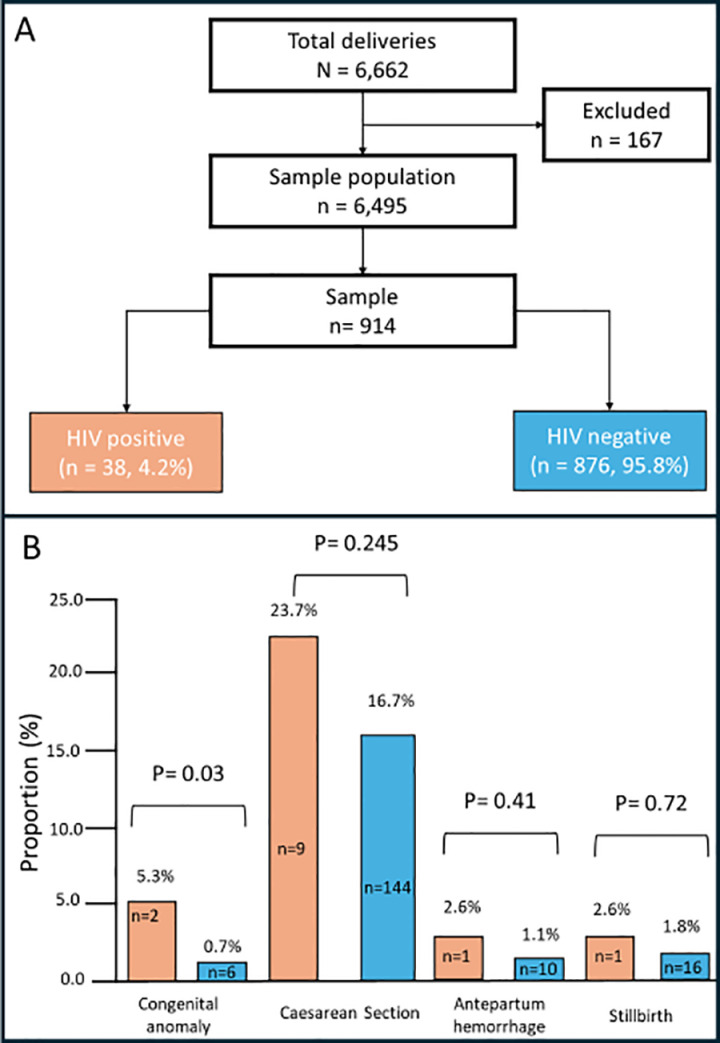
Study flow.

During the three-year study period, there were 6,662 deliveries conducted; 167 were excluded due to either twin deliveries or prehospital birth. A total of 914 charts were systematically sampled, among whom 38 (4.2%) were HIV-positive. All 38 women with HIV were on dolutegravir (DTG)-based ART regimens; 18 were on Tenofovir (TDF)/Lamivudine (3TC)/DTG, while 20 were on AZT/3TC/DTG. Of these, only two participants were initiated on ART during pregnancy. The obstetrics and neonatal outcomes were compared between mothers with and without HIV infection (Fig 1).

### Socio-demographic characteristics

Table 1 summarizes the socio-demographic characteristics of the study participants. The baseline characteristics were comparable. However, a higher proportion of women with HIV were significantly older (26.0 vs 22.0, p < 0.001) and were multiparous (73.7% vs 47.2%, p < 0.001), compared to their counterparts without HIV infection.

### Comparison of obstetric outcomes

There was no statistically significant difference between the groups, with regards to mode of delivery, indications for caesarean section, prevalence of pre-eclampsia, obstetric hemorrhages, perinatal tear, or obstructed labour (Table 2).

### Comparison of neonatal outcomes

Among the study participants, congenital anomalies were found in 0.9% of the participants, with a statistically significantly higher proportion among infants born to women with HIV (5.3%) compared to HIV-unexposed infants (0.7%) (p = 0.003). Among women with HIV, both congenital anomalies documented were cases of anencephaly. In the HIV-negative group, anomalies included a case of gastroschisis, cleft lip and palate, clubbed foot, two hydrocephaly, and one unspecified anomaly noted as gross congenital anomalies (Table 3).

### Multivariable analysis

Infants born to women with HIV had an almost 10-folds higher odds of having a congenital anomaly compared to HIV unexposed infants (adjusted odds ratio (aOR): 9.76 (95% CI: 1.72–55.48, p = 0.010) (Table 4).

**Table 4:** Multivariable analysis for association between HIV and congenital anomaly.

Variable	Congenital Anomaly		Bivariate		Multivariable	
	Yes n (%)	No n (%)	cOR (95% CI)	P – value	aOR (95% CI)	P – value
HIV			8.06 (1.57–41.31)	**0.012**	9.76 (1.72–55.48)	**0.01**
Positive	2 (25.00)	36 (3.97)				
Negative	6 (75.00)	870 (96.03)				
Pre-eclampsia			5.48 (0.45–46.42)	0.118	6.65 (0.72–61.30)	0.094
Yes	1 (12.50)	23 (2.54)				
No	7 (87.50)	883 (97.46)				
Age	22.38	22.18	1.01 (0.90–1.13)	0.09	0.90 (0.77–1.06)	0.195
Parity			3.25 (0.65–16.18)	0.150	5.10 (0.79–32.87)	0.086
Multiparous	6 (75.00)	435 (48.01)				
nulliparous	2 (25.00)	471 (51.99)				

## Discussion

In this comparative study of obstetric and newborn outcomes, we found a markedly increased risk of congenital anomalies among infants born to women living with HIV, with an almost 10-fold elevation in adjusted odds. This finding aligns with and contributes to a growing body of literature suggesting a potential teratogenic risk associated with DTG-based ART, particularly when initiated or taken at the time of conception [16,18,26]. Our cohort exclusively comprised HIV-positive women on DTG-containing regimen reflecting current Ugandan guidelines and thus offers contemporary relevance. The elevated risk of congenital anomalies, particularly neural tube defects (NTDs), is consistent with findings from Botswana’s Tsepamo study, which first raised alarms in May 2018 when an interim analysis revealed a higher prevalence of NTDs among infants born to women who conceived while on DTG (0.94%) compared to other ART regimens [26]. Subsequent surveillance, including a larger dataset released in August 2019, reported five NTDs among 1,683 DTG-exposed pregnancies (0.30%) compared to 15 NTDs among 14,792 pregnancies with other ART exposures (0.10%), resulting in an absolute risk difference of 0.20% (3 vs. 1 per 1,000 deliveries). Importantly, in all DTG-associated cases, ART had commenced more than three months prior to conception, underscoring the significance of preconception exposure. The suspected mechanism for DTG teratogenicity involves interference with folate receptor binding, impairing folate uptake essential for neural tube closure during early embryogenesis [27,28]. Although this pathway is biologically plausible, the clinical magnitude of risk remains a subject of ongoing investigation. Indeed, most infants born to women on DTG do not manifest congenital anomalies, and the overall benefit-risk profile of DTG given its efficacy, tolerability, and low resistance profile continues to support its widespread use. In addition to ART exposure, we must consider other unmeasured but biologically plausible contributors to congenital anomalies. Opportunistic infections such as cytomegalovirus (CMV), toxoplasmosis, and syphilis, which are prevalent in immunocompromised individuals and capable of transplacental transmission, are independently associated with congenital anomalies [5,17]. These infections were not assessed in our study which limits the ability to fully attribute observed anomalies solely to ART exposure. Similarly, data on maternal CD4 + count, viral load suppression, nutritional status (especially folate levels), and ART adherence were unavailable, precluding assessment of their potential confounding effects. Another source of interpretative caution is the wide confidence interval around our effect estimate (aOR 9.76, 95% CI: 1.72–55.48), reflecting statistical imprecision likely stemming from the low frequency of congenital anomalies observed in both groups. This range underscores substantial uncertainty and limits the generalizability of the findings. The observed association, though statistically significant, should be interpreted conservatively until replicated in larger studies with robust control of confounders and more granular data on ART timing and infection status. Additionally, the influence of unmeasured socioeconomic determinants such as maternal education, access to antenatal care, household income, and rural residence may contribute indirectly to adverse birth outcomes by affecting nutrition, health-seeking behaviours, and infection risk. These are important avenues for future research. Previous studies may have found no association between HIV and congenital anomalies because they primarily examined older ART regimens, such as efavirenz or niverapine, prior to the widespread adoption of DTG-based therapy [19,23,29].

Aside from congenital anomalies, our study found no significant differences in maternal outcomes such as caesarean section rates, obstructed labour, perineal tears, postpartum hemorrhage (PPH), and puerperal sepsis. These findings align with studies from Ethiopia [30], Mbarara, Uganda [31], Nigeria [7], and Kenya [32], showing comparable obstetric outcomes between HIV-positive and HIV-negative women in settings where ART is widely accessible. However, other reports from Denmark [14] and the United States [15] have found higher caesarean section rates among HIV-positive women, often attributed to differing clinical guidelines and healthcare capacities.

Beyond congenital anomalies, our study found that neonatal outcomes including stillbirth, Apgar scores, birth asphyxia, birthweight, and NICU admissions did not differ significantly between women with and without HIV infection. These findings on stillbirth are consistent with several studies reporting no significant association with maternal HIV status [9,32,33]. However, evidence from Southern Africa [8,17,19] has documented higher still birth rates among HIV-positive women, often attributed to ART-related complications and underlying maternal health comorbidities. Comparable Apgar scores between the two groups suggest that immediate neonatal wellbeing is largely unaffected by maternal HIV status. This aligns with previous research from Kenya [33] and Nigeria [7,30], although some studies from Nigeria [20] and in India [21] have reported lower Apgar scores among neonates born to HIV-positive mothers, potentially reflecting contextual differences in clinical care and maternal health. The incidence of birth asphyxia was also similar across groups, corroborating findings from Denmark [14] and China [34]. This contrasts with data from Nigerian [20], were higher rates were observed, possibly due to regional disparities in obstetric and neonatal care. While earlier studies have frequently reported lower birth weights among infants of HIV-positive mothers [6–10], our findings showed no significant difference. This supports emerging evidence that effective ART, particularly in the context of integrated antenatal care, can mitigate the risk of intrauterine growth restriction [30,33]. Similarly, NICU admission rates were not elevated among HIV-exposed neonates in our study, aligning with findings from Tanzania [35]. This suggests that optimal management of HIV in pregnancy, particularly with current ART regimens, may contribute to neonatal outcomes comparable to those in HIV-negative populations. In contrast, a study from India [21] reported higher NICU admissions, underscoring the potential influence of healthcare system variability and maternal health status on neonatal outcomes.

### Strength and limitations

This study has several strengths, including a large sample size that enhances statistical power and reliability, as well as a comprehensive evaluation of both maternal and neonatal outcomes. The fact that all HIV-positive mothers were receiving DTG-based ART regimens, a widely adopted ART regimen, adds to the study’s relevance and timeliness.

However, certain limitations should be acknowledged. The low number of observed congenital anomalies limits the ability to draw definitive conclusions or generalize findings regarding their association with maternal HIV status. The study did not assess opportunistic infections, which may act as confounding factors. Additionally, unmeasured variables such as socioeconomic status, nutritional status, and ART adherence could have influenced outcomes. Data on CD4 + count and viral load were not available, which limits assessment of maternal immune status and virologic control. Lastly, reliance on medical records may have introduced risks of incomplete or inaccurate data.

### Implications of the study findings

Given the increased risk of congenital anomalies observed in HIV-positive women receiving DTG-based ART, heightened antenatal surveillance is warranted. As maternal and other neonatal outcomes were found to be largely comparable between HIV-positive and HIV-negative women, these findings provide reassurance regarding the overall safety of ART in pregnancy. Nonetheless, further research is needed to better understand the potential teratogenic risks associated with DTG, particularly in resource-limited settings.

### Future direction

Future investigations should adopt a design that captures detailed data on ART initiation timing, maternal nutritional status, and virologic control. Such efforts would help delineate the factors contributing to the observed congenital anomalies and guide safer ART choices during pregnancy.

## Conclusions

This study demonstrated a significantly increased risk of congenital anomalies among neonates born to women with HIV, all of whom were receiving DTG-based ART. However, other obstetric and newborn outcomes including caesarean sections, labour complications, postpartum haemorrhage, stillbirth, Apgar scores, birth asphyxia, birth weight, and NICU admissions were similar between HIV-positive and HIV-negative women. The observed association with congenital anomalies must be interpreted cautiously due to wide confidence intervals and small number of events.

Based on our findings, we recommend enhanced prenatal monitoring of HIV-positive pregnant women, particularly those on DTG-based ART. This should include detailed screening for congenital anomalies during antenatal visits. Future studies should aim to clarify the role of DTG and other maternal factors in the etiology of congenital anomalies in HIV-exposed pregnancies.

## Supporting information

S1 Dataset. Data set used for analysis.
(XLS)

## Abbreviations

AGH: Anaka General Hospital
AIDS: Acquired Immune Deficiency Syndrome
aOR: Adjusted Odds Ratio
APH: Antepartum Haemorrhage
ART: Anti-Retroviral Therapy
C/S: Caesarean Section
CD4 +: Clusters of Differentiation 4 positive
CI: Confidence Interval
cOR: Crude Odds Ratio
GUREC: Gulu University Research Ethics Committee
HAART: Highly Active Antiretroviral Therapy
HIV: Human Immunodeficiency Virus
LBW: Low Birth Weight
MTCT: Mother To Child Transmission
NICU: Neonatal Intensive Care Unit
PPH: Post-partum haemorrhage
SVD: Spontaneous vaginal Delivery
UNICEF: United Nations Children’s Fund
Vs.: Versus
WHO: World Health Organization.
