# Supplementary material for: Obstetric and newborn outcomes of mothers with and without HIV infection in Anaka general hospital in Northern Uganda
Source: PLoS One. 2025 Jun 17;20(6):e0326322. doi: 10.1371/journal.pone.0326322 (PMC12173179; doi:10.1371/journal.pone.0326322)
Supplement: Attachment — Submitted filename: PLOS.docx [file pone.0326322.s002.docx]

PLOS ONE

**Obstetric and Newborn Outcomes of Mothers with and without HIV Infection in a General Hospital in Northern Uganda**

--Manuscript Draft--

| **Manuscript Number:** | PONE-D-24-35993 |
| --- | --- |
| **Article Type:** | Research Article |
| **Full Title:** | Obstetric and Newborn Outcomes of Mothers with and without HIV Infection in a General Hospital in Northern Uganda |
| **Short Title:** | HIV and delivery outcomes in a General Hospital in Northern Uganda |
| **Corresponding Author:** | Jolly Joe Lapat, MBChB, MMED Anaka General Hospital  Gulu, Uganda UGANDA |
| **Keywords:** | HIV; Antiretroviral Therapy; Obstetrics Outcomes; Neonatal Outcomes; Congenital Anomalies. |
| **Abstract:** | Background: Women of reproductive age comprise over half of the 39 million people living with HIV worldwide. For two decades, policies have recommended ART for all pregnant women with HIV, improving maternal and newborn health. However, concerns about adverse pregnancy outcomes remain. This study compared obstetric and neonatal outcomes among women with and without HIV infection in a rural hospital in northern Uganda.  Methods: A comparative analysis of obstetric and newborn outcomes among women with and without HIV infection who delivered at Anaka General Hospital (July 2020 - June 2023) was conducted. Data were abstracted from maternity and neonatal registers. Associations between adverse outcomes were assessed using Chi-square or Fisher’s exact test for categorical variables and independent sample t-test for numerical variables. Multivariable logistic regression controlled for other independent variables. A p-value <0.05 was considered statistically significant.  Results: Of 914 participants, 38 (4.2%) were HIV-positive. The overall mean age was  22.2 years. Women with HIV were significantly older (26.0 vs 22.0, p < 0.001) and more likely to be multiparous (73.7% vs 47.2%, p < 0.001). The odds of congenital anomalies were nearly 10 times higher among women with HIV (aOR = 9.76, 95% CI 1.72 – 55.48). No significant differences were found in obstetric outcomes (cesarean rates, labor complications, perineal tears, postpartum hemorrhage, puerperal sepsis) or neonatal outcomes (stillbirth rates, Apgar scores, birth asphyxia, birth weight, NICU admissions).  Conclusion: We found a comparable obstetric and newborn outcome among women with and without HIV infection. However, the odds of congenital anomalies were higher among infants of women with HIV. We recommend enhanced prenatal screening and specialized care to women with HIV to mitigate the risk of congenital anomalies in their infants. |
| **Order of Authors:** | Jolly Joe Lapat, MBChB, MMED |
|  | Jimmyy Opee, MBChB, MMED |
|  | James Okello, MBChB, MPH |
|  | Nixson Oyoo, MBChB |
|  | Christopher Wanican, BNS |
|  | Gloria Becky Labong, RM |
|  | Daniel Ebbs, DO, MS, EMT-P, FAAP |
|  | Felix Bongomin, MBChB, MSc, MMED |
| **Additional Information:** |  |
| **Question** | **Response** |
| **Financial Disclosure** | The author(s) received no specific funding for this work. |

| Enter a financial disclosure statement that describes the sources of funding for the work included in this submission. Review the [submission guidelines](http://journals.plos.org/plosone/s/submission-guidelines#loc-financial-disclosure-statement) for detailed requirements. View published research articles from [*PLOS ONE*](https://journals.plos.org/plosone/) for specific examples.  This statement is required for submission and **will appear in the published article** if the submission is accepted. Please make sure it is accurate. |  |
| --- | --- |
| **Funded studies**  Enter a statement with the following details:   - Initials of the authors who received each award - Grant numbers awarded to each author - The full name of each funder - URL of each funder website - Did the sponsors or funders play any role in the study design, data collection and analysis, decision to publish, or preparation of the manuscript? |  |
| Did you receive funding for this work? |  |
| **Competing Interests**  Use the instructions below to enter a competing interest statement for this submission. On behalf of all authors, disclose any [competing interests](http://journals.plos.org/plosone/s/competing-interests) that could be perceived to bias this work—acknowledging all financial support and any other relevant financial or non- financial competing interests.  This statement is required for submission and **will appear in the published article** if the submission is accepted. Please make sure it is accurate and that any funding sources listed in your Funding Information later in the submission form are also declared in your Financial Disclosure statement. | The authors have declared that no competing interests exist |

| View published research articles from  [*PLOS ONE*](http://journals.plos.org/plosone/) for specific examples. |  |
| --- | --- |
| **NO authors have competing interests**  Enter: *The authors have declared that no competing interests exist.*  **Authors with competing interests**  Enter competing interest details beginning with this statement:  *I have read the journal's policy and the authors of this manuscript have the following competing interests: [insert competing interests here]* |  |
| * typeset |  |
| **Ethics Statement**  Enter an ethics statement for this submission. This statement is required if the study involved:   - Human participants - Human specimens or tissue - Vertebrate animals or cephalopods - Vertebrate embryos or tissues - Field research   Write "N/A" if the submission does not require an ethics statement.  General guidance is provided below. Consult the [submission guidelines](http://journals.plos.org/plosone/s/submission-guidelines#loc-guidelines-for-specific-study-types) for detailed instructions. **Make sure that all information entered here is included in the Methods section of the manuscript.** | Ethics approval and consent to participate were secured prior to the study's initiation. The Gulu Research Ethics Committee granted approval for the study (Ref GUREC- 2023-586), including a waiver of consent. Consent waswaived off since this was a record review |

| **Format for specific study types**  **Human Subject Research (involving human participants and/or tissue)**   - Give the name of the institutional review board or ethics committee that approved the study - Include the approval number and/or a statement indicating approval of this research - Indicate the form of consent obtained (written/oral) or the reason that consent was not obtained (e.g. the data were analyzed anonymously)   **Animal Research (involving vertebrate animals, embryos or tissues)**   - Provide the name of the Institutional Animal Care and Use Committee (IACUC) or other relevant ethics board that reviewed the study protocol, and indicate whether they approved this research or granted a formal waiver of ethical approval - Include an approval number if one was obtained - If the study involved *non-human primates*, add *additional details* about animal welfare and steps taken to ameliorate suffering - If anesthesia, euthanasia, or any kind of animal sacrifice is part of the study, include briefly which substances and/or methods were applied   **Field Research**  Include the following details if this study involves the collection of plant, animal, or other materials from a natural setting:   - Field permit number - Name of the institution or relevant body that granted permission |  |
| --- | --- |
|  |  |
| **Data Availability**  Authors are required to make all data underlying the findings described fully available, without restriction, and from the time of publication. PLOS allows rare exceptions to address legal and ethical concerns. See the [PLOS Data Policy](http://journals.plos.org/plosone/s/data-availability) and [FAQ](http://journals.plos.org/plosone/s/data-availability#loc-faqs-for-data-policy) for detailed information. | Yes - all data are fully available without restriction |

| A Data Availability Statement describing where the data can be found is required at submission. Your answers to this question constitute the Data Availability Statement and **will be published in the article**, if accepted. |  |
| --- | --- |
| **Important:** Stating ‘data available on request from the author’ is not sufficient. If your data are only available upon request, select ‘No’ for the first question and explain your exceptional situation in the text box. |  |
| Do the authors confirm that all data underlying the findings described in their manuscript are fully available without restriction? |  |
| **Describe where the data may be found in full sentences. If you are copying our sample text, replace any instances of XXX with the appropriate details.**   - If the data are **held or will be held in a public repository**, include URLs, accession numbers or DOIs. If this information will only be available after acceptance, indicate this by ticking the box below. For example: *All XXX files are available from the XXX database (accession number(s) XXX, XXX.*). - If the data are all contained **within the manuscript and/or Supporting Information files**, enter the following: *All relevant data are within the manuscript and its Supporting Information files.* - If neither of these applies but you are able to provide **details of access elsewhere**, with or without limitations, please do so. For example:   *Data cannot be shared publicly because of [XXX]. Data are available from the XXX Institutional Data Access / Ethics Committee (contact via XXX) for researchers who meet the criteria for access to confidential data.*  *The data underlying the results presented in the study are available from (include the name of the third party* | All relevant data are within the manuscript and its Supporting Information files. |

| *and contact information or URL).*   - This text is appropriate if the data are owned by a third party and authors do not have permission to share the data.   * typeset |  |
| --- | --- |
| Additional data availability information: |  |

Manuscript	[Click here to access/download;Manuscript;Lapat et. al. HIV and](https://www2.cloud.editorialmanager.com/pone/download.aspx?id=37343332&guid=82513938-6284-4208-9c39-5151029d85d1&scheme=1)[<https://www2.cloud.editorialmanager.com/pone/download.aspx?id=37343332&guid=82513938-6284-4208-9c39-5151029d85d1&scheme=1> delivery outcome manuscript .docx](https://www2.cloud.editorialmanager.com/pone/download.aspx?id=37343332&guid=82513938-6284-4208-9c39-5151029d85d1&scheme=1)

# 1 Obstetric and Newborn Outcomes of Mothers with and without HIV Infection in a

2 **General Hospital in Northern Uganda**

3

4 Jolly Joe Lapat ^1,2*^, Jimmyy Opee^1,3^, James Okello^2^, Nixson Oyoo^1,4^, Christopher
5 Wanican^2^, Gloria Becky Labong^2^, Daniel S. Ebbs^5^, Felix Bongomin ^1,6,7^

6

# 7 Author details

8 1. Department of Public Health, Faculty of Medicine, P.O BOX 166, Gulu, Uganda
9 2. Anaka General Hospital, Nwoya District Local Government, P. O. Box 1033, Gulu,
10 Uganda
11 3. Department of Reproductive Health, Faculty of Medicine, Gulu University, P.O.
12 BOX 166, Gulu, Uganda
13 4. Kitgum General Hospital, Kitgum District Local Government, P. O. Box 28,
14 Kitgum, Ugnada
15 5. Section of Critical Care Medicine, Department of Paediatrics, Yale University,
16 New Haven, Connecticut, United States of America
17 6. Department of Medical Microbiology and Immunology, Faculty of Medicine, Gulu
18 University, P. O. BOX, 166, Gulu, Uganda
19 7. Department of Internal Medicine, Gulu Regional Referral Hospital, Gulu, Uganda
20 Correspondence
21 Dr Jolly Joe Lapat
22 Department of Obstetrics and Gynecology
23 Anaka General Hospital, Nwoya District Local Government, P. O. Box 1033, Gulu,
24 Uganda.

# 25 Abstract

26 **Background:** Women of reproductive age comprise over half of the 39 million people
27 living with HIV worldwide. For two decades, policies have recommended ART for all
28 pregnant women with HIV, improving maternal and newborn health. However, concerns
29 about adverse pregnancy outcomes remain. This study compared obstetric and neonatal
30 outcomes among women with and without HIV infection in a rural hospital in northern
31 Uganda.
32 **Methods:** A comparative analysis of obstetric and new born outcomes among women with
33 and without HIV infection who delivered at Anaka General Hospital (July 2020 - June
34 2023) was conducted. Data were abstracted from maternity and neonatal registers.
35 Associations between adverse outcomes were assessed using Chi-square or Fisher’s exact
36 test for categorical variables and independent sample t-test for numerical variables.
37 Multivariable logistic regression controlled for other independent variables. A p-value
38 <0.05 was considered statistically significant.
39 **Results:** Of 914 participants, 38 (4.2%) were HIV-positive. The overall mean age was 22.2
40 years. Women with HIV were significantly older (26.0 vs 22.0, p < 0.001) and more likely
41 to be multiparous (73.7% vs 47.2%, p < 0.001). The odds of congenital anomalies were
42 nearly 10 times higher among women with HIV (aOR = 9.76, 95% CI 1.72 – 55.48). No
43 significant differences were found in obstetric outcomes (cesarean rates, labor
44 complications, perineal tears, postpartum hemorrhage, puerperal sepsis) or neonatal
45 outcomes (stillbirth rates, Apgar scores, birth asphyxia, birth weight, NICU admissions).
46 **Conclusion:** We found a comparable obstetric and newborn outcome among women with
47 and without HIV infection. However, the odds of congenital anomalies were higher among
48 infants of women with HIV. We recommend enhanced prenatal screening and specialized
49 care to women with HIV to mitigate the risk of congenital anomalies in their infants.
50 **Keywords:** HIV, Antiretroviral Therapy, Maternal Outcomes, Neonatal Outcomes,
51 Congenital Anomalies, Dolutegravir, Uganda

# 52 Introduction

53 Women of reproductive age account for more than half of the 39 million people living with
54 human immunodeficiency virus (HIV) worldwide (1). Most women with HIV are in the
55 reproductive age group and are capable of passing this infection to their babies. Most (90%)
56 of the infections in children aged under 15 years are from maternally-acquired HIV
57 infection. For two decades the World Health Organization (WHO) and UNICEF have
58 recommended that all pregnant women with HIV commence anti-retroviral therapy (ART)
59 during pregnancy regardless of CD4+ cell count and continue it for life (2). This
60 recommendation has led to more women with HIV living healthy lives and becoming
61 pregnant while on ART. Over 1.5 million women with HIV are estimated to give birth
62 annually (3). According to the Joint United Nations Programme on HIV/AIDS (UNAIDS)
63 2023 global report, between 64 – 98% of pregnant women with HIV have access to ART
64 globally (1).
65 ART has significantly reduced maternal morbidity and mortality (1,4,5), yet concerns
66 persist regarding delivery outcomes for women with HIV compared to their HIV-negative
67 counter parts. Studies indicate that women with HIV are 2- to 3- folds more likely to face
68 adverse pregnancy outcomes, attributed to direct effect of HIV, complications from ART,
69 and socioeconomic challenges (6–8). Furthermore, women with HIV are at a higher risk
70 for various pregnancy complications, including preterm labor (7,9), maternal anemia (10),
71 puerperal sepsis (11,12), post-partum hemorrhage (11–13), intrauterine growth restriction
72 (IUGR) (14), and an increased likelihood of cesarean sections (14,15)
73 Neonates born to HIV-positive mothers often experience adverse outcomes such as birth
74 defects (16–18), stillbirths (17,19), low Apgar score (7,9) low birthweight (20), Neonatal
75 Intensive Care (NICU) admission (21), and higher neonatal mortality rates (7,19). While
76 Highly active Anti retro viral Therapy (HAART) effectively reduces the risk of mother-to-
77 child transmission (MTCT) of HIV and improves maternal health (1,12), certain regimens,
78 particularly those containing zidovudine (AZT) and dolutegravir (DTG), are associated
79 with an increased risk of congenital anomalies (22,23). Therefore, while ART benefits in
80 reducing mother to child transmission (MTCT) and enhancing maternal health are well-
81 documented, it may be associated with adverse obstetric and neonatal outcomes, influenced
82 by socioeconomic status and rural residence, underscores the need for ongoing research.
83 There are limited studies on the outcomes of women with HIV in Uganda, highlighting the
84 need for further research/evaluation. This study aimed to compare the maternal and
85 newborn outcomes of women with and without HIV infection who gave birth at Anaka
86 General Hospital, Northern Uganda.

# 87 Methods

88 **Study design and setting**
89 This study was a hospital-based retrospective records review study of deliveries conducted
90 at the maternity ward of Anaka General Hospital, Nwoya District, Northern Uganda from
91 July 2020 to June 2023. Anaka General Hospital is the only government-owned hospital
92 under governance of Nwoya District Local Government. The Hospital is the major referral
93 facility for the district. The clientele includes patients from Nwoya as well as neighboring
94 districts. The hospital has an average of 2000 deliveries conducted annually. All pregnant
95 mothers in labour and those with pregnancy complications are admitted to the maternity
96 ward, which also houses a Neonatal Intensive Care Unit for managing sick newborns. All
97 HIV positive women are enrolled into care at the Mother Baby care clinic at first ANC
98 contact, Viral Load counts is done every three months. CD4+ test is not routinely done.
99 And all clients are started on a DTG containing regimen according to policy (24)

# 100 Study population.

101 The study population consisted of all deliveries from Anaka General Hospital between July
102 2020 and June 2023. Excluded were charts of women who delivered before arrival and
103 twin pregnancies due to their higher risk of adverse outcomes.

# 104 Sample size estimation

105 Sample size was calculated with the Kish Leslie formula established in 1965 for sample
106 size determination, incorporating the double proportion formula (25). By utilizing a critical
107 value of 1.96 at a significance level of 5% and considering a margin of error of 0.05 along
108 with a statistical power of 80%, a sample size of 914 was deemed adequate for detecting a
109 minimum odds difference of 2.0 in low birth weight compared to a previous study (8)

# 110 Sampling strategy.

111 During the study period, there were a total of 6,662 deliveries. We excluded 167 charts
112 because they were either twin delivery or births before arrival at the hospital. Systematic
113 sampling technique was used to sample charts from the integrated maternity register. The
114 sampling intervals were determined by calculating “K” values, using formula K = N/n,
115 where “N” is the total deliveries and “n” is the calculated sample size. K = 6495/914 ≈ 7.
116 The sampling intervals was 7^th^ interval. The first chart was selected randomly.

# 117 Data collection

118 The process of data collection, encompassing both records retrieval and data extraction,
119 spanned a period of two months, commencing on January 3, 2024, and concluding on
120 February 29, 2024. Data was collected from patient files using a structured data extraction
121 tool. Two midwives, trained by the principal investigator, served as research assistants.
122 They were briefed on the study’s objectives, data collection procedures, and how to
123 accurately complete the extraction tool. Sample charts identified from the integrated
124 maternity register were listed, including names and in-patient numbers. Subsequently,
125 charts of sampled mothers and babies admitted to the NICU were retrieved from the records
126 section. Data extraction was performed from these files using a structured tool. The authors
127 had no access to information that could identify individual participants during or after data
128 collection.

# 129 Study variables.

130 Our outcome variables were grouped as obstetric or newborn. The obstetric outcome
131 variables included occurrence of mode of delivery (categorized as vaginal delivery or
132 caesarean section), preeclampsia, defined as hypertension and proteinuria (++) using urine
133 dipstick, ante partum haemorrhage, episiotomy performed, perineal tear, defined as any
134 tear that required repair, post-partum haemorrhage and maternal death. The newborn
135 outcome variables included delivery status (alive or dead), birth weight, Apgar score at 1
136 and 5 minutes, presence of congenital anomalies, admission to NICU and neonatal death.
137 The data extraction tool captured data on independent variables including socio-
138 demographic characteristics and HIV status. The main socio-demographic factors included
139 age, area of residence and referral status. Medical factors captured focused on HIV status.
140 The obstetric factors included gravidity, parity, and gestation age at delivery, stage of
141 labour at admission and induction of labor.

# 142 Data management and analysis

143 The raw data was thoroughly checked for accuracy and completeness before being coded
144 and entered into an Excel spreadsheet. Analysis was conducted using STATA software
145 version 17 SE, with all tests being two-tailed at 95% confidence interval and significance
146 level set at P < 0.05. Baseline characteristics of participants were summarized with the
147 corresponding P-values indicating distribution disparities between cohorts. Association
148 between categorical variables and HIV status was analyzed using Chi-square test or
149 Fisher’s exact test where appropriate. Association between numerical variables and HIV
150 status was assessed using independent student’s t-test. Level of statistical significance was
151 set at p < 0.05. Outcome variables that had significant association with HIV status were
152 taken to multivariate logistic regression to adjust for other independent variables. Results
153 are presented as adjusted odds ratio (aOR) with the corresponding 95 % confidence interval
154 and *p –* values (Table 1 - 3).

# 155 RESULTS

## 156 Study enrollment

157 During the 3-year study period, there were 6,662 deliveries conducted; 167 were excluded
158 due to twin delivery or prehospital birth. A total of 914 were systematically sampled, out
159 of which there were 38 (4.2%) HIV positive women. All 38 women with HIV were on
160 Dolutegravir (DTG)-based ART regimen; 18 were on Tenofovir (TDF)/Lamivudine
161 (3TC)/DTG, while 20 were on AZT/3TC/DTG. Of these, only two participants were
162 initiated on ART during pregnancy. The obstetrics and neonatal outcomes were compared
163 between mothers with and without HIV infection (Figure 1).

164

# 165 Figure 1: Study flow

## 166 Socio-demographic characteristics

167 Table 1 summarizes the socio-demographic characteristics of the study participants. The
168 baseline characteristics were comparable. However, a higher proportion of women with
169 HIV were significantly older (26.0 vs 22.0, p < 0.001) and were multiparous (73.7% vs
170 47.2%, p < 0.001), compared to their counterparts without HIV infection.

# 171 Table 1: Comparison of socio-demographics characteristics

| **Characteristics** | **Total N (%)** | **With HIV n (%)** | **Without HIV n (%)** | **P value** |
| --- | --- | --- | --- | --- |
| **Age** (mean, sd) | 22.2 | 26.0 | 22.0 | **< 0.001** |
| **Address** |  |  |  |  |
| Urban | 421 (46.2) | 15 (39.5) | 406 (46.5) | 0.395 |
| Rural | 490 (53.8) | 23 (60.5) | 467 (53.5) |  |
| **Parity**  Multiparous | 441(48.3) | 28 (73.7) | 413 (47.2) | **0.001** |
| Nulliparous | 473 (51.7) | 10 (26.3) | 463 (52.8) |  |
| **Referral in**  Yes | 109 (11.9) | 4 (10.5) | 105 (12.0) | 0.786 |
| No | 805 (88.1) | 34 (89.5) | 771 (88.0) |  |
| **Labor onset** |  |  |  |  |

0.919

| Induced | 26 (2.9) | 1 (2.6) | 25 (2.9) |
| --- | --- | --- | --- |
| Spontaneous  **Stage of labor** | 370 (97.1) | 37 (97.4) | 833 (97.1) |

|  | Not in labor | 111 (12.1) | 6 (15.8) | 105 (12.0) | 0.743 |
| --- | --- | --- | --- | --- | --- |
|  | Latent phase | 250 (27.4) | 8 (21.1) | 242 (27.6) |  |
|  | Active phase | 412 (45.1) | 17 (44.7) | 359 (45.1) |  |
|  | Second stage | 141 (15.4) | 7 (18.4) | 134 (15.3) |  |
| 172 |  |  |  |  |  |

## 173 Comparison of Obstetric outcomes

174 There was no statistically significant difference between the groups, with regards to mode
175 of delivery, indications for caesarean section, prevalence of pre-eclampsia, obstetric
176 hemorrhages, perinatal tear, or obstructed labour (Table 2).

# 177 Table 2: Comparison of obstetric outcomes

| Variable | | | Total N (%) | HIV Positive  n (%) | HIV Negative  n (%) | P value |
| --- | --- | --- | --- | --- | --- | --- |
| **Mode of delivery** | | |  |  |  |  |
| Caesarean | | | 153 (16.7) | 9 (23.7) | 144 (16.4) | 0.245 |
| Vaginal | | | 761 (83.3) | 29 (76.3) | 732 (83.6) |  |
| **Indication of C/S**  Prolong/obstructed | | | 38 (25.2) | 1 (11.1) | 37 (26.0) | 0.234 |
| Fetal distress | | | 19 (12.6) | 1 (11.1) | 18 (12.7) |  |
| Previous C/S | | | 45 (29.8) | 6 (66.7) | 39 (27.5) |  |
| Contracted pelvis | | | 25 (16.5) | 1 (11.1) | 24 (16.9) |  |
| Pre-eclampsia | | | 9 (6.0) | 0 (0.0) | 9 (6.3) |  |
| Others | | | 15 (9.9) | 0 (0.0) | 15 (10.6) |  |
| **Type of caesarean**  Elective | | | 10 (6.6) | 0 (0.0) | 10 (7.0) | 0.412 |
| Emergent | | | 142 (93.4) | 9 (100.0) | 133 (93.0) |  |
| **Pre-eclampsia** | | |  |  |  |  |
|  | | Yes | 24 (2.6) | 0 (0.0) | 24 (2.7) | 0.619 |
|  | | No | 890 (97.4) | 38 (100.0) | 852 (97.3) |  |
| **Ante-partum** | |  |  |  |  |  |
| **hemorrhage** | |  | 11 (1.2) | 1 (2.6) | 10 (1.1) | 0.410 |
| Yes | | | 903 (98.8) | 37 (97.4) | 866 (98.9) |  |
| No | | |  |  |  |  |
| **Obstructed/prolonged** | | |  |  |  |  |
| Yes | | | 31 (3.4) | 2 (5.3) | 29 (3.3) | 0.516 |
| No | | | 882 (96.6) | 36 (94.7) | 846 (96.7) |  |
| **Perineal tear** | |  |  |  |  |  |
| Tear | | | 119 (13.0) | 5 (13.2) | 114 (13.0) | 0.979 |
| No tear | | | 795 (87.0) | 33 (86.8) | 762 (87.0) |  |
| **Episiotomy** | |  |  |  |  |  |
| Performed | | | 44 (4.8) | 1 (2.6) | 43 (4.9) | 0.521 |
| Not performed | | | 870 (95.2) | 38 (97.4) | 833 (95.1) |  |
| **Post-partum** | | |  |  |  |  |
| **hemorrhage** | | | 12 (1.3) | 0 (0.0) | 12 (1.4) | 1.00 |
| Yes | | | 902 (98.7) | 38 (100.0) | 864 (98.6) |  |
| No | | |  |  |  |  |
| **Puerperal sepsis** | | | | | | |
|  |  | Yes | 1 (0.1) | 0 (0.0) | 1 (0.1) | 1.00 |
|  |  | No | 456 (99.9) | 38 (100.0) | 875 (99.9) |  |
|  | **Hospitalization** | **days** | 2 (1, 18) | 2 (1, 8) | 1 (1, 18) | 0.433 |
|  | (median, range) |  |  |  |  |  |
| 178 |  |  |  |  |  |  |

## 179 Comparison of neonatal outcomes

180 Among the study participants, congenital anomalies were found in 0.9% of the participants,
181 with a statistically significantly higher proportion among infants born to women with HIV
182 (5.3%) compared to HIV-unexposed infants (0.7%) (p = 0.003). Among women with HIV,
183 both congenital anomalies documented were cases of anencephaly. In the HIV-negative
184 group, anomalies included gastroschisis (1), cleft lip and palate (1), clubbed foot (1),
185 hydrocephaly (2), and one unspecified anomaly noted as gross congenital anomalies (Table
186 3).

# 187 Table 3: Comparison of neonatal outcome

| Variable |  | | Total N (%) | HIV positive  n (%) | HIV negative  n (%) | P value |
| --- | --- | --- | --- | --- | --- | --- |
| **Status** |  | |  |  |  |  |
|  | Stillbirth | | 17 (1.9) | 1(2.6) | 16 (1.8) | 0.719 |
|  | Live birth | | 897 (98.1) | 37 (97.4) | 860 (98.2) |  |
| **Apgar 1 minute** | | |  |  |  |  |
| < 7 | | | 77 (8.4) | 3 (7.9) | 74 (8.5) | 0.924 |
| 7 – 10 | | | 836 (91.6) | 35 (92.1) | 801 (91.5) |  |
| **Apgar 5 minute** | | |  |  |  |  |
| < 7 | | | 25 (2.6) | 1 (2.6) | 24 (2.7) | 0.967 |
| 7 – 10 | | | 889 (97.4) | 37 (97.4) | 851 (97.3) |  |
| **Baby sex** | | |  |  |  |  |
| Male | | | 456 (49.9) | 21 (55.3) | 435 (49.7) | 0.499 |
| Female | | | 458 (50.1) | 17 (44.7) | 441 (50.3) |  |
| **Birth asphyxia** | | | | | | |
|  | | Yes | 92 (10.3) | 3 (8.11) | 89 (10.4) | 0.660 |
|  | | No | 805 (89.7) | 34 (91.9) | 771 (89.6) |  |
| **Birth weight** | |  | 2.9 | 2.76 | 2.93 | 0.032 |
| **Anomaly** | |  |  |  |  |  |
|  | | Yes | 8 (0.9) | 2 (5.3) | 6 (0.7) | **0.003** |
|  | | No | 905 (99.1) | 36 (94.7) | 869 (99.3) |  |
| **NICU admission** | | | | | | |
| Yes | | | 104 (11.59) | 6 (16.2) | 98 (11.4) | 0.370 |
| No | | | 793 (88.41) | 31 (83.8) | 762 (88.6) |  |
| **Discharged alive** | | | | | | |

188

No Yes

44 (4.81)

870 (95.84)

2 (5.3)

36 (94.7)

42 (4.8)

834 (95.2)

0.895

## 189 Multivariable analysis

190 Infants born to women with HIV had an almost 10-folds higher odds of having a congenital
191 anomaly compared to HIV unexposed infants (adjusted odds ratio (aOR): 9.76 (95% CI:
192 1.72 – 55.48, p = 0.010) (Table 4).

# 193 Table 4. Multivariable analysis for association between HIV and congenital anomaly

**Variable	Congenital Anomaly	Bivariate	Multivariable**

Yes n (%)	No n (%)	cOR (95% CI)	P – value	aOR (95% CI)	P – value

# HIV

| Positive | 2 (25.00) | 36 (3.97) | 8.06 (1.57 – 41.31) | **0.012** | 9.76 (1.72 – 55.48) | **0.010** |
| --- | --- | --- | --- | --- | --- | --- |
| Negative | 6 (75.00) | 870 (96.03) |  |  |  |  |
| **Pre- eclampsia** | 1 (12.50) | 23 (2.54) | 5.48 (0.45 – 46.42) | 0.118 | 6.65 (0.72 – 61.30) | 0.094 |
| Yes No  **Age** | 7 (87.50)  22.38 | 883 (97.46)  22.18 | 1.01 (0.90 – 1.13) | 0.09 | 0.90 (0.77 – 1.06) | 0.195 |
| **Parity**  Multiparous | 6 (75.00) | 435 (48.01) | 3.25 (0.65 – 16.18) | 0.150 | 5.10 (0.79 – 32.87) | 0.086 |
| nulliparous | 2 (25.00) | 471 (51.99) |  |  |  |  |

194

195	**Discussion**

196

197 In this study comparing obstetric and newborn outcomes, we found that infants born to
198 women with HIV have an almost 10-fold higher odds of having a congenital anomaly
199 compared to infants born to women without HIV. This risk maybe associated with ART,
200 particularly DTG-based ART regimens, which all the women with HIV were on. Findings
201 from our study aligns with existing literature showing a heightened risk of congenital
202 anomalies linked to DTG use (16,18,26) For instance, a large national birth outcome
203 surveillance study in Botswana reported a significantly higher risk of neural tube defects
204 with preconception DTG exposure compared to HIV-negative women (26). DTG is
205 thought to exert teratogenic effects by inhibiting folate receptors, disrupting folate transport
206 essential for proper neurulation during critical developmental days (27,28). In addition to
207 ART exposure, opportunistic infections such as cytomegalovirus (CMV), toxoplasmosis,
208 and syphilis may increase the risk of congenital anomalies in HIV-positive women, as these
209 infections can cross the placental barrier and harm the fetus, increasing the risk for
210 congenital anomalies (17)(5). Prior studies may have found no association between HIV
211 and congenital anomalies due to their focus on older ART regimens like Efavirenz or
212 Niverapine, before widespread DTG use (19,23,29)
213 This study found that obstetric outcomes, including caesarean section rates, obstructed or
214 prolonged labor, perineal tears, postpartum hemorrhage (PPH), and puerperal sepsis, were
215 comparable between women with and without HIV. Conflicting data remains on caesarean
216 section rates between HIV-positive and negative women (10, 14-15, 30). In particular
217 contrast to this study, (10,14,15) found higher rates for caesarean sections likely due to
218 local healthcare practices and resources. Labor complications were similar between groups,
219 consistent with studies showing comparable outcomes with effective ART management
220 (30), though an Ethiopian study (31) suggests higher complications for HIV-positive
221 women, possibly due to healthcare access differences. The absence of significant
222 differences in PPH and puerperal sepsis is consistent with findings from Mbarara, Uganda
223 (32), and Nigeria (7), but contrasts with studies (11,13), reporting higher rates in HIV-
224 positive women with limited ART and perinatal care access.
225 Apart from congenital anomalies, our found nebron outcomes such as stillbirth, Apgar
226 score, birth asphyxia, birthweight, and NICU admissions were similar between HIV-
227 positive and HIV-negative women. Our findings on stillbirth align with studies showing
228 no significant difference between groups (9,30,33), though some research in Southern
229 Africa reported higher rates among HIV-positive women (8,17,19) attributed to possible
230 complications related to ART and maternal health conditions. Apgar scores were
231 comparable, suggesting that immediate neonatal outcomes are not significantly impacted
232 by maternal HIV status which is similar to findings of studies in Kenya (33) and Nigeria
233 (7,30). although some studies from Nigeria (20) and in India (21) have reported lower
234 scores in infants born to HIV-positive mothers. Birth asphyxia rates were similar between
235 groups, consistent with studies from Denmark (14) and China (34) but differing from a
236 Nigerian study (20) that found higher rates, possibly due to regional disparities. Most
237 studies indicate lower birth weights in infants of HIV-positive mothers (6–10), but our
238 study's finding of no significant difference aligns with research suggesting effective ART
239 can mitigate this risk (30,33). NICU admission rates were similar to findings from Tanzania
240 (35), indicating effective ART management results in comparable outcomes, in contrast to
241 a study from India (21), that reported higher NICU admissions for infants born to HIV-
242 positive mothers.

## 243 Strength and limitations

244 This study's strengths include a substantial sample size, enhancing statistical power and
245 reliability, and a comprehensive assessment of maternal and neonatal outcomes. Since all
246 the HIV mothers were on DTG-based ART regimens, a widely used ART regimens, makes
247 this study particularly relevant and timely.
248 However, the study also had limitations. The low number of congenital anomalies limits
249 the study's ability to draw definitive conclusions or generalize the association between HIV
250 status and the risk of anomalies. The study did not assess opportunistic infections, which
251 could confound the results. Uncontrolled factors like socioeconomic status, nutritional
252 status, and ART adherence might influence outcomes. CD4+ and viral load information
253 were not obtained. Additionally, reliance on medical records introduces a risk of
254 incomplete or inaccurate reporting.

# 255 Implications of the study findings

256 The implications of this study indicate that healthcare providers should be vigilant and
257 enhance monitoring of HIV-positive pregnant women on DTG for congenital anomalies,
258 include routine screening for opportunistic infections such as CMV, toxoplasmosis and
259 syphilis, and provide comprehensive counseling on ART risks and benefits during
260 pregnancy. Additionally, clinical guidelines should be updated to address DTG risks in
261 pregnancy, resources should be allocated for comprehensive prenatal care for HIV-positive
262 women, and medical record-keeping should be improved for accurate outcome reporting.

# 263 Future direction

264 Future research should conduct larger longitudinal studies on the long-term effects of DTG
265 exposure. It should compare the impacts of different ART regimens on pregnancy and
266 neonatal outcomes, evaluate confounding factors like socioeconomic and nutritional status
267 and ART adherence, opportunistic infections and expand studies to diverse geographic
268 locations for broader generalizability.

# 269 Conclusions.

270 In this study there is nearly 10-fold increase in odds of congenital anomaly among neonates
271 delivered by HIV infected mother. However, all other newborn and obstetric outcomes are
272 comparable between mothers with and without HIV infection. This study recommends
273 enhance monitoring of HIV-positive pregnant women on DTG for congenital anomalies,
274 include routine screening for opportunistic infections as part of routine care. We also
275 recommend that further research is essential to deepen our understanding of the
276 relationship between HIV and congenital anomalies and to develop effective strategies to
277 mitigate these risks.

# 278 Abbreviations

| 279 | AGH | Anaka General Hospital |
| --- | --- | --- |
| 280 | AIDS | Acquired Immune Deficiency Syndrome |
| 281 | aOR | Adjusted Odds Ratio |
| 282 | APH | Antepartum Haemorrhage |
| 283 | ART | Anti-Retroviral Therapy |
| 284 | C/S | Caesarean Section |
| 285 | CD4+ | Clusters of Differentiation 4 positive |
| 286 | CI | Confidence Interval |
| 287 | cOR | Crude Odds Ratio |
| 288 | GUREC | Gulu University Research Ethics Committee |
| 289 | HAART | Highly Active Antiretroviral Therapy |
| 290 | HIV | Human Immunodeficiency Virus |
| 291 | LBW | Low Birth Weight |
| 292 | MTCT | Mother To Child Transmission |
| 293 | NICU | Neonatal Intensive Care Unit |
| 294 | PPH | Post-partum haemorrhage |

295 SVD	Spontaneous vaginal Delivery
296 UNICEF	United Nations Children’s Fund
297 Vs.	Versus
298 WHO	World Health Organization

# 299 Acknowledgements

300 We extend our sincere gratitude to the Department of Public Health at Gulu University and
301 its esteemed lecturers for their invaluable support throughout this study. Our heartfelt
302 thanks go to the administration of Anaka General Hospital, the Office of the District Health
303 Officer, and the administration of Nwoya District Local Government for their endorsement
304 and facilitation of this research. We also acknowledge the significant contributions of our
305 research assistants, Acen Lucy and Ahirwe Clemensia, whose dedication and effort in data
306 collection were instrumental to the success of this study.

# 307 Author contributions

308 JJL, JO, and FB contributed to the conceptualization and design of the study. JJL performed
309 the formal data analysis. JJL drafted the manuscript. JO, JO, NO, CW, GBL, DSE and FB
310 critically reviewed and revised the manuscript for key content. JJL prepared the final
311 manuscript. All authors read and approved the final manuscript.

# 312 Funding

313 The research received no specific grant from any funding agency in the public, commercial
314 or non-for-profit sectors.

# 315 Data availability

316 The research data and materials used to support the research findings of this study are
317 available on request from the corresponding author.

# 318 Declarations

319 Ethics approval and consent to participate were secured prior to the study's initiation. The
320 Gulu Research Ethics Committee granted approval for the study (Ref GUREC-2023-586),
321 including a waiver of consent. Additionally, administrative clearance was obtained from
322 the Chief Administrative Officer of Nwoya District Local Government to proceed with the
323 research.

# 324 Consent for publication.

325 Not applicable

# 326 Competing interests

327 The authors declare that there are no interests regarding the publication of this paper.

# 328 References

329 1.	UNAIDS. UNAIDS Global AIDS update 2022 [Internet]. 2022 [cited 2024
330 Apr 13]. Available from:
331 https:[/www.unaids.org/sitess/default/files/media_asset/2022-global-](http://www.unaids.org/sitess/default/files/media_asset/2022-global-)
332 aids-update-summary_en.pdf

333

334 2.	WHO. The use of Antiretroviral drugs for treating and preventing HIV
335 Infection [Internet]. 2016 [cited 2024 Jul 13]. Available from:
336 https:[/www.who.int/publications/i/item/9789241549684](http://www.who.int/publications/i/item/9789241549684)

337

338 3.	Stringer EM, Kendall MA, Lockman S, Campbell TB, Nielsen-Saines K,
339 Sawe F, et al. Pregnancy outcomes among HIV-infected women who
340 conceived on antiretroviral therapy. PLoS One. 2018;13(7):e0199555. 341
342 4.	Calvert C, Marston M, Slaymaker E, Crampin AC, Price AJ, Klein N, et al.
343 Direct maternal deaths attributable to HIV in the era of antiretroviral
344 therapy: evidence from three population-based HIV cohorts with verbal
345 autopsy. Aids. 2020;34(9):1397–405.

346

347 5.	Smith CT, Megli C, Chappell CA. Infectious Diseases in Pregnancy.
348 Obstetric Anesthesia and Uncommon Disorders. 2023;367. 349
350 6.	Portwood C, Sexton H, Kumarendran M, Brandon Z, Kirtley S, Hemelaar
351 J. Adverse perinatal outcomes associated with antiretroviral therapy in
352 women living with HIV: A systematic review and meta-analysis. Front
353 Med (Lausanne). 2023;9:924593.

354

355 7.	Ezechi OC, Oladele DA, Kalejaiye OO, Oke BO, Ohwodo HO, Adu RA, et al.
356 Pregnancy, obstetric and neonatal outcomes in HIV positive Nigerian
357 women. Afr J Reprod Health. 2013;17(3):160–8. 358
359 8.	Tukei VJ, Hoffman HJ, Greenberg L, Thabelo R, Nchephe M, Mots’oane T,
360 et al. Adverse pregnancy outcomes among HIV-positive women in the
361 era of universal antiretroviral therapy remain elevated compared with
362 HIV-negative women. Pediatr Infect Dis J. 2021;40(9):821–6. 363
364 9.	Worku WZ, Azale T, Ayele TA, Mekonnen DK. Effects of HIV infection on
365 pregnancy outcomes among women attending antenatal care in referral
366 hospitals of the Amhara Regional State, Ethiopia: a prospective cohort
367 study. Int J Womens Health. 2022;1405–23. 368
369 10.	Ikpim EM, Edet UA, Bassey AU, Asuquo OA, Inyang EE. HIV infection in
370 pregnancy: maternal and perinatal outcomes in a tertiary care hospital
371 in Calabar, Nigeria. Trop Doct. 2016;46(2):78–86. 372
373 11.	Calvert C, Ronsmans C. HIV and the risk of direct obstetric
374 complications: a systematic review and meta-analysis. PLoS One.
375 2013;8(10):e74848.

376

377 12.	Sebitloane HM, Moodley D. The impact of highly active antiretroviral
378 therapy on obstetric conditions: a review. European Journal of
379 Obstetrics & Gynecology and Reproductive Biology. 2017;210:126–31. 380
381 13.	Sebitloane HM, Moodley J. Maternal and obstetric complications among
382 HIV-infected women treated with highly active antiretroviral treatment
383 at a Regional Hospital in Durban, South Africa. Niger J Clin Pract.
384 2017;20(11):1360–7.

385

386 14.	Ørbaek M, Thorsteinsson K, Moseholm Larsen E, Katzenstein TL,
387 Storgaard M, Johansen IS, et al. Risk factors during pregnancy and birth‐
388 related complications in HIV‐positive versus HIV‐negative women in
389 Denmark, 2002–2014. HIV Med. 2020;21(2):84–95.

390

391 15.	Arab K, Spence AR, Czuzoj-Shulman N, Abenhaim HA. Pregnancy
392 outcomes in HIV-positive women: a retrospective cohort study. Arch
393 Gynecol Obstet. 2017;295:599–606.

394

395 16.	Pereira GFM, Kim A, Jalil EM, Fonseca FF, Shepherd BE, Veloso VG, et al.
396 Dolutegravir and pregnancy outcomes in women on antiretroviral
397 therapy in Brazil: a retrospective national cohort study. Lancet HIV.
398 2021;8(1):e33–41.

399

400 17.	Shava E, Moyo S, Zash R, Diseko M, Dintwa EN, Mupfumi L, et al. Brief
401 report: high rates of adverse birth outcomes in HIV and syphilis
402 coinfected women in Botswana. JAIDS Journal of Acquired Immune
403 Deficiency Syndromes. 2019;81(5):e135–40. 404

405	18.	Raesima MM, Ogbuabo CM, Thomas V, Forhan SE, Gokatweng G, Dintwa

406	E, et al. Dolutegravir use at conception—additional surveillance data

407	from Botswana. New England Journal of Medicine. 2019;381(9):885–7. 408

409	19.	Santosa WB, Staines-Urias E, Tshivuila-Matala COO, Norris SA,

410	Hemelaar J. Perinatal outcomes associated with maternal HIV and

411	antiretroviral therapy in pregnancies with accurate gestational age in

412	South Africa. Aids. 2019;33(10):1623–33.

413

414	20.	Adewole ND, Okechukwu AA, Offiong RA. Pregnancy Outcome of HIV

415	Positive Mothers on Antiretroviral Therapy in a Tertiary Health

416	Institution in Abuja, Nigeria. 417

418	21.	Dadhwal V, Sharma A, Khoiwal K, Deka D, Sarkar P, Vanamail P.

419	Pregnancy outcomes in HIV-infected women: experience from a tertiary

420	care center in India. Int J MCH AIDS. 2017;6(1):75. 421

422	22.	Dugdale CM, Ciaranello AL, Bekker LG, Stern ME, Myer L, Wood R, et al.

423	Risks and benefits of dolutegravir-and efavirenz-based strategies for

424	South African women with HIV of child-bearing potential: a modeling

425	study. Ann Intern Med. 2019;170(9):614–25.

426

427	23.	Ford N, Mofenson L, Shubber Z, Calmy A, Andrieux-Meyer I, Vitoria M, et

428	al. Safety of efavirenz in the first trimester of pregnancy: an updated

429	systematic review and meta-analysis. Aids. 2014;28:S123–31. 430

431	24.	MOH. Consolidated Guidelines for the Prevention and Treatment of HIV

432	in Uganda [Internet]. 2018 [cited 2023 Apr 3]. Available from:

433	https://platform.who.int/docs/default-source/mca-documents/policy-

434	documents/guidelines/UGA-RH-43-01-GUIDELINE-2018-eng-Final-

435	Uganda-Guidlines.pdf

436

437	25.	Kish L. Sampling organizations and groups of unequal sizes. Am Sociol

438	Rev. 1965;564–72.

439

440	26.	Zash R, Makhema J, Shapiro RL. Neural-tube defects with dolutegravir

441	treatment from the time of conception. New England Journal of

442	Medicine. 2018;379(10):979–81.

443

444	27.	Cabrera RM, Souder JP, Steele JW, Yeo L, Tukeman G, Gorelick DA, et al.

445	The antagonism of folate receptor by dolutegravir: developmental

446	toxicity reduction by supplemental folic acid. Aids. 2019;33(13):1967–

447	76.

448

449	28.	Gilmore JC, Hoque MT, Dai W, Mohan H, Dunk C, Serghides L, et al.

450	Interaction between dolutegravir and folate transporters and receptor

451	in human and rodent placenta. EBioMedicine. 2022;75. 452

453	29.	de Tejada BM. Birth defects after exposure to efavirenz-based

454	antiretroviral therapy at conception/first trimester of pregnancy: a

455	multicohort analysis. JAIDS Journal of Acquired Immune Deficiency

456	Syndromes. 2019;80(3):316–24.

457

458	30.	Adenmosun IO, Adesina O. Comparison of Pregnancy and Neonatal

459	Outcomes Amongst HIV Positive Women and HIV Negative Women at

460	the University College Hospital Ibadan, Nigeria–A Cross-sectional Study.

461	African Journal of Biomedical Research. 2020;23(SE1):53–7. 462

463	31.	Lolaso T, Oljira L, Dessie Y, Gebremedhin M, Wakgari N. Adverse birth

464	outcome and associated factors among newborns delivered in public

465	health institutions, Southern Ethiopia. East African Journal of Health

466	and Biomedical Sciences. 2019;3(2):35–44.

467

468	32.	Bebell LM, Ngonzi J, Siedner MJ, Muyindike WR, Bwana BM, Riley LE, et

469	al. HIV Infection and risk of postpartum infection, complications and

470	mortality in rural Uganda. AIDS Care. 2018;30(8):943–53. 471

472	33.	Mugo C, Nduati R, Osoro E, Nyawanda BO, Mirieri H, Hunsperger E, et al.

473	Comparable pregnancy outcomes for HIV-uninfected and HIV-infected

474	women on antiretroviral treatment in Kenya. J Infect Dis.

475	2022;226(4):678–86.

476

477	34.	Li H, Liu J, Tan D, Huang G, Zheng J, Xiao J, et al. Maternal HIV infection

478	and risk of adverse pregnancy outcomes in Hunan province, China: a

479	prospective cohort study. Medicine. 2020;99(8):e19213. 480

481	35.	Rebnord T, Mmbaga BT, Sandøy IF, Lie RT, Mchome B, Mahande MJ, et

482	al. Time trends in perinatal outcomes among HIV-positive pregnant

483	women in Northern Tanzania: A registry-based study. PLoS One.

484	2023;18(8):e0289740.

485

data

General comments

First, I would like to thank you for inviting me to review this **manuscript**. It is known that study of outcomes HIV infected pregnant women is vital issue globally for the last 2-3 decades after emerging of the virus. Many researches were done on this throughout the world.

- The paper manuscript is well organized except some errors which I commented on it
- Please add variables which are not included in previous studies like viral load and CD4 count and others.
- Try to show the gaps of previous studies and significance of this study.
- Minor revision is required, the manuscript is well addressed.

[Click here to access/download](https://www2.cloud.editorialmanager.com/pone/download.aspx?id=37271122&guid=f123cda6-3310-4b7d-ba95-bf397f2dfee7&scheme=1)

[**Supporting Information** Data for analysis.dta](https://www2.cloud.editorialmanager.com/pone/download.aspx?id=37271122&guid=f123cda6-3310-4b7d-ba95-bf397f2dfee7&scheme=1)
