# Supplementary material for: Obstetric and newborn outcomes of mothers with and without HIV infection in Anaka general hospital in Northern Uganda
Source: PLoS One. 2025 Jun 17;20(6):e0326322. doi: 10.1371/journal.pone.0326322 (PMC12173179; doi:10.1371/journal.pone.0326322)
Supplement: Attachment — Submitted filename: Response to reviewers.docx [file pone.0326322.s004.docx]

Lapat Jolly Joe,

Obstetrician and Gynaecologist,

Anaka General Hospital,

P. O. Box 1033, Gulu, Uganda.

[lapatjj5@gmail.com](mailto:lapatjj5@gmail.com)

4^th^ – May – 2025

**Editorial Team**

PLOS ONE

Dear Editorial team,

Rebuttal to Editor and Reviewers

**Ref: Rebuttal letter that responds to each point raised by the academic editor and reviewer(s)**

We sincerely thank the editor and reviewers for their constructive comments and valuable feedback. We have thoroughly revised the manuscript to address all concerns raised. Below, we provide a detailed point-by-point response.

**Editor’s Comments and Responses**

1. Manuscript Format and Language: We have formatted the manuscript according to the PLOS ONE style requirements using the provided templates.

**Response**: The file names were updated accordingly. The manuscript has also been professionally edited for English grammar, punctuation, and clarity by a qualified English editor.

2. English Language Editing:

**Response:** The manuscript was edited. Both a clean version and a tracked-changes version will be submitted as required.

3. Ethics Statement:

**Response:** The ethics statement has been moved to the Methods section, as per the journal guidelines.

4. Data Availability Statement:

**Response:** We have now added a Data Availability Statement. The minimal data set used for analysis will be uploaded as a Supporting Information file. It includes the data behind all tables and results, excluding any personally identifying information.

5. Supporting Information File Issue:

**Response:** The previous file has been revised and replaced. Captions for Supporting Information files have been included at the end of the manuscript, and in-text citations have been updated accordingly.

**Reviewer #1 Comments and Author Responses**

**Concern 1:** while systematic sampling is the strength, it is not clear whether all medical charts were complete and whether those mothers who received care at multiple facilities were included.

**Response:** We have clarified that only complete charts with full delivery and neonatal outcome records were included. Mothers who delivered at Anaka General Hospital but received antenatal care elsewhere were still included, as long as their delivery data were complete and documented.

**Concern 2:** Confounding variables. Although mentioned in limitation section, such important confounders like CD4 count, viral load, and opportunistic infections that were not collected require stronger discussion of their potential role in the risk of birth defects.

**Response**: We agree and have expanded our limitations and discussion to acknowledge that the absence of these variables restricts our ability to control for potential confounding. We now emphasize that this is a key area for future research.

**Concern 3:** Statistical analysis: sample size of 2 for congenital anomalies is important but very small for this outcome. The use of an adjusted odds ratio (aOR) with such few cases may not be statistically robust. Consider using alternative approaches like exact logistic regression.

**Response:** We acknowledge this limitation and have elaborated on the uncertainty in our results. We retained logistic regression due to its interpretability and because it remains standard in similar studies, but added caution in interpretation and suggested exact logistic regression as an alternative for future studies.

**Concern 4 (i).** The authors should be careful in the discussion section not to imply causality which may not be a valid conclusion given the small number of anomalies observed and the study design

**Response:** We have revised the Discussion section to emphasize the observational nature of the study and avoided causal language.

**Concern 4 (ii):** Also, the authors mention the 2018 Tsepamo study in Botswana (N=426) stating that similar to this study, it reported significantly higher risk of congenital anomalies in HIV-positive women on DTG. However, the authors failed to mention the 2019 updated results of Tsepamo study with much larger sample (1683 pregnancies exposed to DTG) which showed an updated and recalculated lower prevalence of 0.3 versus 0.9 in the initial report.

**Response:** This has been corrected. The updated 2019 Tsepamo study with a recalculated lower prevalence (0.3%) has been included and discussed to reflect current understanding.

**Reviewer #2 Comments and Author Responses**

**Concern 1:** The paper manuscript is well organized except some errors which I commented on it.

**Response:** The manuscript has undergone comprehensive to address grammar, clarity, and structure.

**Concern 2:** Please add variables which are not included in previous studies like viral load and CD4 count and others.

**Response:** We have acknowledged the absence of these variables as a limitation. Unfortunately, this information was not routinely recorded in delivery charts. Future prospective studies are suggested.

**Concern:** Try to show the gaps of previous studies and significance of this study.

**Response:** The introduction and conclusion have been revised to highlight the gaps in data on HIV and pregnancy outcomes in Uganda, particularly regarding DTG use.

All reviewer and editorial concerns have been addressed. The revised manuscript reflects improved scientific clarity, transparency about limitations, and alignment with journal guidelines. We are grateful for the opportunity to revise our work and hope it now meets the standards for publication.

Sincerely,

**Lapat Jolly Joe**, on behalf of all co-authors
