# Supplementary material for: Obstetric and newborn outcomes of mothers with and without HIV infection in Anaka general hospital in Northern Uganda
Source: PLoS One. 2025 Jun 17;20(6):e0326322. doi: 10.1371/journal.pone.0326322 (PMC12173179; doi:10.1371/journal.pone.0326322)
Supplement: Peer review history

# Author comment: Author response to Decision Letter 0

**DOI:** 10.1371/journal.pone.0326322.r001

**Transfer Alert**

This paper was transferred from another journal. As a result, its full editorial history (including decision letters, peer reviews and author responses) may not be present.

28 Aug 2024

# Peer review: Decision Letter 0

**DOI:** 10.1371/journal.pone.0326322.r002

**Authors:** Keralem Bishaw

Dear Dr. Lapat,

Thank you for submitting your manuscript to PLOS ONE. After careful consideration, we feel that it has merit but does not fully meet PLOS ONE’s publication criteria as it currently stands. Therefore, we invite you to submit a revised version of the manuscript that addresses the points raised during the review process.

Please submit your revised manuscript by May 08 2025 11:59PM. If you will need more time than this to complete your revisions, please reply to this message or contact the journal office at plosone@plos.org . When you're ready to submit your revision, log on to https://www.editorialmanager.com/pone/ and select the 'Submissions Needing Revision' folder to locate your manuscript file.

- A rebuttal letter that responds to each point raised by the academic editor and reviewer(s). You should upload this letter as a separate file labeled 'Response to Reviewers'.
- A marked-up copy of your manuscript that highlights changes made to the original version. You should upload this as a separate file labeled 'Revised Manuscript with Track Changes'.
- An unmarked version of your revised paper without tracked changes. You should upload this as a separate file labeled 'Manuscript'.

If you would like to make changes to your financial disclosure, please include your updated statement in your cover letter. Guidelines for resubmitting your figure files are available below the reviewer comments at the end of this letter.

If applicable, we recommend that you deposit your laboratory protocols in protocols.io to enhance the reproducibility of your results. Protocols.io assigns your protocol its own identifier (DOI) so that it can be cited independently in the future. For instructions see: https://journals.plos.org/plosone/s/submission-guidelines#loc-laboratory-protocols . Additionally, PLOS ONE offers an option for publishing peer-reviewed Lab Protocol articles, which describe protocols hosted on protocols.io. Read more information on sharing protocols at https://plos.org/protocols?utm_medium=editorial-email&utm_source=authorletters&utm_campaign=protocols .

We look forward to receiving your revised manuscript.

Kind regards,

Keralem Anteneh Bishaw, Msc.

Academic Editor

PLOS ONE

Journal Requirements:

When submitting your revision, we need you to address these additional requirements.

1. Please ensure that your manuscript meets PLOS ONE's style requirements, including those for file naming. The PLOS ONE style templates can be found at

https://journals.plos.org/plosone/s/file?id=wjVg/PLOSOne_formatting_sample_main_body.pdf and

https://journals.plos.org/plosone/s/file?id=ba62/PLOSOne_formatting_sample_title_authors_affiliations.pdf .

2. We suggest you thoroughly copyedit your manuscript for language usage, spelling, and grammar. If you do not know anyone who can help you do this, you may wish to consider employing a professional scientific editing service.

The American Journal Experts (AJE) (https://www.aje.com/) is one such service that has extensive experience helping authors meet PLOS guidelines and can provide language editing, translation, manuscript formatting, and figure formatting to ensure your manuscript meets our submission guidelines. Please note that having the manuscript copyedited by AJE or any other editing services does not guarantee selection for peer review or acceptance for publication.

Upon resubmission, please provide the following:

 The name of the colleague or the details of the professional service that edited your manuscript

 A copy of your manuscript showing your changes by either highlighting them or using track changes (uploaded as a *supporting information* file)

 A clean copy of the edited manuscript (uploaded as the new *manuscript* file).

3. Your ethics statement should only appear in the Methods section of your manuscript. If your ethics statement is written in any section besides the Methods, please move it to the Methods section and delete it from any other section. Please ensure that your ethics statement is included in your manuscript, as the ethics statement entered into the online submission form will not be published alongside your manuscript.

4. We note that your Data Availability Statement is currently as follows: [Add Data Availability statement here]

Please confirm at this time whether or not your submission contains all raw data required to replicate the results of your study. Authors must share the “minimal data set” for their submission. PLOS defines the minimal data set to consist of the data required to replicate all study findings reported in the article, as well as related metadata and methods (https://journals.plos.org/plosone/s/data-availability#loc-minimal-data-set-definition).

For example, authors should submit the following data:

- The values behind the means, standard deviations and other measures reported;

- The values used to build graphs;

- The points extracted from images for analysis.

Authors do not need to submit their entire data set if only a portion of the data was used in the reported study.

If your submission does not contain these data, please either upload them as Supporting Information files or deposit them to a stable, public repository and provide us with the relevant URLs, DOIs, or accession numbers. For a list of recommended repositories, please see https://journals.plos.org/plosone/s/recommended-repositories.

If there are ethical or legal restrictions on sharing a de-identified data set, please explain them in detail (e.g., data contain potentially sensitive information, data are owned by a third-party organization, etc.) and who has imposed them (e.g., an ethics committee). Please also provide contact information for a data access committee, ethics committee, or other institutional body to which data requests may be sent. If data are owned by a third party, please indicate how others may request data access.

5. We are unable to open your Supporting Information file [File Name]. Please kindly revise as necessary and re-upload.

6. Please include captions for your Supporting Information files at the end of your manuscript, and update any in-text citations to match accordingly. Please see our Supporting Information guidelines for more information: http://journals.plos.org/plosone/s/supporting-information.

Additional Editor Comments (if provided):

Date: March 21, 2025

Manuscript ID: PONE-D-24-35993 "Obstetric and Newborn Outcomes of Mothers with and without HIV Infection in a General Hospital in Northern Uganda".

General comment:

• The manuscript is with a full grammatical error, awkward sentences, spacing and punctuation. It needs English language editors.

• Issues in the methodology part should addressed.

• Discussion and conclusion of the study along with component the paper should be improved.

• The document should paraphrased.

• Arrange the manuscript based on the journal guideline.

Abstract part

Background: This is a copy and paste of thee introduction part “Women of reproductive age comprise over half of the 39 million people living with HIV worldwide.” Try to paraphrase it.

Method: under this section try to incorporate all methodology component like sample size sampling technique and state the design (method) you applied.

Result section: try to mention only the main outcome of the study only. No need of considering such information “he overall mean age was 22.239 years.” Since, it is not the primary objective of the study.

Conclusion: make the recommendation in line with the finding. You state the general recommendation.

Sampling strategy

Try to omit unnecessary punctuation and clearly state which types of random sampling technique you applied to the first sample specifically.

Sample calculation: please state how to allocate sample for two groups.

Data collection

It expected from to state clearly from where data extraction checklist developed or (adapted), its contents and data quality control method considered in addition to providing training for the data collectors.

Discussion

It is a full spelling and punctuation error and grammatical error. For example line “225”. Apart from congenital anomalies, our found nebron outcome, “Instead of stating such information “CD4+ and viral load information were not obtained. Additionally, reliance on medical records introduces a risk of incomplete or inaccurate reporting.” Clearly state the limitation of the study.

Implications of the study finding

Try to make in line with the finding of the study Based on your finding instead on stating general recommendation such as “routine screening for opportunistic infections such as CMV, toxoplasmosis and syphilis, and provide comprehensive counseling on ART risks and benefits during pregnancy”

Finally, reported the manuscript using strobe reporting checklist for observation studies.

[Note: HTML markup is below. Please do not edit.]

Reviewers' comments:

Reviewer's Responses to Questions

**Comments to the Author**

1. Is the manuscript technically sound, and do the data support the conclusions?

Reviewer #1: Yes

Reviewer #2: Yes

**********

2. Has the statistical analysis been performed appropriately and rigorously?

Reviewer #1: Yes

Reviewer #2: Yes

**********

3. Have the authors made all data underlying the findings in their manuscript fully available?

The PLOS Data policy

Reviewer #1: Yes

Reviewer #2: Yes

**********

4. Is the manuscript presented in an intelligible fashion and written in standard English?

Reviewer #1: Yes

Reviewer #2: Yes

**********

Reviewer #1: The study addresses an important global health issue - the impact of HIV and ART on pregnancy and neonatal outcomes. These days when dolutegravir-based regimens are widespread, the issue of its potential link to congenital anomalies is concerning. The study's retrospective design and substantial sample size are the study's strengths.

However, several aspects of the manuscript could be improved in terms of clarity, methodology, analysis and discussion.

The major concerns are:

1. Study design and sampling. While systematic sampling technique is the strength, its not clear whether all medical charts included were complete and whether those mothers who received care at multiple facilities were included.

2. Confounding variables. Although mentioned in limitation section, such important confounding variables, i.e., opportunistic infections, socioeconomic factors, and CD4 and viral load data that were not collected require stronger discussion of their potential role in the risk of birth defects.

3. Statistical analysis. Sample size of 2 for congenital anomalies is important but very small for this outcome. the use of an adjusted odds ratio (aOR) with such few cases may not be statistically robust. Consider using alternative statistical approaches like exact logistic regression.

Also, confidence intervals (i.e., aOR for congenital anomalies: 1.72-55.48) speaks for considerable uncertainty in the risk estimates. Discussion should be provided as it affects generalizability and reliability of the findings.

4. Discussion. The authors should be careful in the discussion section not to imply causality which may not be a valid conclusion given the small number of anomalies observed and the study design. Also, the authors mention the 2018 Tsepamo study in Botswana (N=426) stating that similar to this study, it reported significantly higher risk of congenital anomalies in HIV-positive women on DTG. However, the authors failed to mention the 2019 updated results of Tsepamo study with much larger sample (1683 pregnancies exposed to DTG) which showed an updated and recalculated lower prevalence of 0.3 versus 0.9 in the initial report.

Conclusion: this manuscript presents important findings contributing to the understanding of neonatal and maternal outcomes in HIV-positive women, particularly in relation to risks associated with dolutegravir. However, improvements in methodology, statistical analysis and discussion would strengthen this manuscript.

Reviewer #2: � The paper manuscript is well organized except some errors which I commented on it

� Please add variables which are not included in previous studies like viral load and CD4 count and others.

� Try to show the gaps of previous studies and significance of this study.

� Minor revision is required, the manuscript is well addressed.

**********

what does this mean? ). If published, this will include your full peer review and any attached files.

If you choose “no”, your identity will remain anonymous but your review may still be made public.

**Do you want your identity to be public for this peer review?** For information about this choice, including consent withdrawal, please see our Privacy Policy

Reviewer #1: No

Reviewer #2: **Yes: ** Endinew Beka Mehiretie

**********

[NOTE: If reviewer comments were submitted as an attachment file, they will be attached to this email and accessible via the submission site. Please log into your account, locate the manuscript record, and check for the action link "View Attachments". If this link does not appear, there are no attachment files.]

While revising your submission, please upload your figure files to the Preflight Analysis and Conversion Engine (PACE) digital diagnostic tool, https://pacev2.apexcovantage.com/ . PACE helps ensure that figures meet PLOS requirements. To use PACE, you must first register as a user. Registration is free. Then, login and navigate to the UPLOAD tab, where you will find detailed instructions on how to use the tool. If you encounter any issues or have any questions when using PACE, please email PLOS at figures@plos.org . Please note that Supporting Information files do not need this step.

Attachment Submitted filename: PLOS.docx

# Author comment: Author response to Decision Letter 1

**DOI:** 10.1371/journal.pone.0326322.r003

5 May 2025

Editor’s Comments and Responses

1. Manuscript Format and Language: We have formatted the manuscript according to the PLOS ONE style requirements using the provided templates.

Response: The file names were updated accordingly. The manuscript has also been professionally edited for English grammar, punctuation, and clarity by a qualified English editor.

2. English Language Editing:

Response: The manuscript was edited. Both a clean version and a tracked-changes version will be submitted as required.

3. Ethics Statement:

Response: The ethics statement has been moved to the Methods section, as per the journal guidelines.

4. Data Availability Statement:

Response: We have now added a Data Availability Statement. The minimal data set used for analysis will be uploaded as a Supporting Information file. It includes the data behind all tables and results, excluding any personally identifying information.

5. Supporting Information File Issue:

Response: The previous file has been revised and replaced. Captions for Supporting Information files have been included at the end of the manuscript, and in-text citations have been updated accordingly.

Reviewer #1 Comments and Author Responses

Concern 1: while systematic sampling is the strength, it is not clear whether all medical charts were complete and whether those mothers who received care at multiple facilities were included.

Response: We have clarified that only complete charts with full delivery and neonatal outcome records were included. Mothers who delivered at Anaka General Hospital but received antenatal care elsewhere were still included, as long as their delivery data were complete and documented.

Concern 2: Confounding variables. Although mentioned in limitation section, such important confounders like CD4 count, viral load, and opportunistic infections that were not collected require stronger discussion of their potential role in the risk of birth defects.

Response: We agree and have expanded our limitations and discussion to acknowledge that the absence of these variables restricts our ability to control for potential confounding. We now emphasize that this is a key area for future research.

Concern 3: Statistical analysis: sample size of 2 for congenital anomalies is important but very small for this outcome. The use of an adjusted odds ratio (aOR) with such few cases may not be statistically robust. Consider using alternative approaches like exact logistic regression.

Response: We acknowledge this limitation and have elaborated on the uncertainty in our results. We retained logistic regression due to its interpretability and because it remains standard in similar studies, but added caution in interpretation and suggested exact logistic regression as an alternative for future studies.

Concern 4 (i). The authors should be careful in the discussion section not to imply causality which may not be a valid conclusion given the small number of anomalies observed and the study design

Response: We have revised the Discussion section to emphasize the observational nature of the study and avoided causal language.

Concern 4 (ii): Also, the authors mention the 2018 Tsepamo study in Botswana (N=426) stating that similar to this study, it reported significantly higher risk of congenital anomalies in HIV-positive women on DTG. However, the authors failed to mention the 2019 updated results of Tsepamo study with much larger sample (1683 pregnancies exposed to DTG) which showed an updated and recalculated lower prevalence of 0.3 versus 0.9 in the initial report.

Response: This has been corrected. The updated 2019 Tsepamo study with a recalculated lower prevalence (0.3%) has been included and discussed to reflect current understanding.

Reviewer #2 Comments and Author Responses

Concern 1: The paper manuscript is well organized except some errors which I commented on it.

Response: The manuscript has undergone comprehensive to address grammar, clarity, and structure.

Concern 2: Please add variables which are not included in previous studies like viral load and CD4 count and others.

Response: We have acknowledged the absence of these variables as a limitation. Unfortunately, this information was not routinely recorded in delivery charts. Future prospective studies are suggested.

Concern: Try to show the gaps of previous studies and significance of this study.

Response: The introduction and conclusion have been revised to highlight the gaps in data on HIV and pregnancy outcomes in Uganda, particularly regarding DTG use.

Attachment Submitted filename: Response to reviewers.docx

# Editor report: Decision Letter 1

**DOI:** 10.1371/journal.pone.0326322.r004

**Authors:** Keralem Bishaw

Obstetric and Newborn Outcomes of Mothers with and without HIV Infection in Anaka General Hospital in Northern Uganda

PONE-D-24-35993R1

Dear Dr. Lapat,

We’re pleased to inform you that your manuscript has been judged scientifically suitable for publication and will be formally accepted for publication once it meets all outstanding technical requirements.

Within one week, you’ll receive an e-mail detailing the required amendments. When these have been addressed, you’ll receive a formal acceptance letter and your manuscript will be scheduled for publication.

An invoice will be generated when your article is formally accepted. Please note, if your institution has a publishing partnership with PLOS and your article meets the relevant criteria, all or part of your publication costs will be covered. Please make sure your user information is up-to-date by logging into Editorial Manager at Editorial Manager®  and clicking the ‘Update My Information' link at the top of the page. If you have any questions relating to publication charges, please contact our Author Billing department directly at authorbilling@plos.org.

If your institution or institutions have a press office, please notify them about your upcoming paper to help maximize its impact. If they’ll be preparing press materials, please inform our press team as soon as possible -- no later than 48 hours after receiving the formal acceptance. Your manuscript will remain under strict press embargo until 2 pm Eastern Time on the date of publication. For more information, please contact onepress@plos.org.

Kind regards,

Keralem Anteneh Bishaw, Msc.

Academic Editor

PLOS ONE

Additional Editor Comments (optional):

I appreciate the author’s efforts in addressing the reviewers’ and editors’ concerns thoroughly. While the retrospective nature of the study imposes certain limitations, including the inability to incorporate some key variables, the findings offer valuable insights to the scientific community. The finding of this study highlights the need for further investigation through higher-level studies, such as prospective cohort or experimental designs.

Despite the noted limitations, we believe the manuscript makes a meaningful contribution to the field and recommend its acceptance for publication in its current form.

Reviewers' comments:

# Editor report: Acceptance letter

**DOI:** 10.1371/journal.pone.0326322.r005

**Authors:** Keralem Bishaw

PONE-D-24-35993R1

PLOS ONE

Dear Dr. Lapat,

I'm pleased to inform you that your manuscript has been deemed suitable for publication in PLOS ONE. Congratulations! Your manuscript is now being handed over to our production team.

At this stage, our production department will prepare your paper for publication. This includes ensuring the following:

* All references, tables, and figures are properly cited

* All relevant supporting information is included in the manuscript submission,

* There are no issues that prevent the paper from being properly typeset

You will receive further instructions from the production team, including instructions on how to review your proof when it is ready. Please keep in mind that we are working through a large volume of accepted articles, so please give us a few days to review your paper and let you know the next and final steps.

Lastly, if your institution or institutions have a press office, please let them know about your upcoming paper now to help maximize its impact. If they'll be preparing press materials, please inform our press team within the next 48 hours. Your manuscript will remain under strict press embargo until 2 pm Eastern Time on the date of publication. For more information, please contact onepress@plos.org.

If we can help with anything else, please email us at customercare@plos.org.

Thank you for submitting your work to PLOS ONE and supporting open access.

Kind regards,

PLOS ONE Editorial Office Staff

on behalf of

Dr. Keralem Anteneh Bishaw

Academic Editor

PLOS ONE
